# Retraction Note: VEGF as a potential molecular target in periodontitis: a meta-analysis and microarray data validation

**DOI:** 10.1186/s12950-024-00380-3

**Published:** 2024-03-15

**Authors:** Bo Ren, Que Feng, Shan He, Yanfeng Li, Jiadong Fan, Guangquan Chai, Le Liu, Haiyun Liu, Chunhao Yang, Yingdi Wang, Huihui Liu, Huanyue Liu, Yafan Song

**Affiliations:** Department of Stomatology, the Fourth Medical Center, Chinese PLA General Hospital, No. 51 Fucheng Road, 100048 Beijing, Haidian District China

**Retraction Note: Journal of Inflammation (2021) 18:18**

10.1186/s12950-021-00281-9

The Editors-in-Chief have retracted this article after concerns were raised about the data reported. The two datasets used in this analysis, GSE10334 and GSE16134, were each generated from the same archive of healthy and diseased gingival tissues, and as such there is a significant sampling overlap between the two. Furthermore, both datasets contain multiple samples per patient. The analytical approach employed by the authors in this paper does not appear to account for these limitations. The Editors-in-Chief no longer have confidence in the reliability of the article’s results and conclusions.

The authors did not respond to correspondence from the publisher about this retraction.

